# PRMT1 and PRMT4 Regulate Oxidative Stress-Induced Retinal Pigment Epithelial Cell Damage in SIRT1-Dependent and SIRT1-Independent Manners

**DOI:** 10.1155/2015/617919

**Published:** 2015-10-25

**Authors:** Dong-Il Kim, Min-Jung Park, Joo-Hee Choi, In-Seon Kim, Ho-Jae Han, Kyung-Chul Yoon, Sang-Woo Park, Min-Young Lee, Ki-Seok Oh, Soo-Hyun Park

**Affiliations:** ^1^College of Veterinary Medicine, Chonnam National University, Gwangju 500-757, Republic of Korea; ^2^Life Science Institute, University of Michigan, Ann Arbor, MI 48109, USA; ^3^Departments of Molecular & Integrative Physiology and Medicine, University of Michigan, Ann Arbor, MI 48109, USA; ^4^Division of Applied Bioscience and Biotechnology, Institute of Environmentally-Friendly Agriculture, College of Agriculture and Life Sciences, Chonnam National University, Gwangju 500-757, Republic of Korea; ^5^College of Veterinary Medicine and Research Institute for Veterinary Science and BK21 PLUS Creative Veterinary Research Center, Seoul National University, Seoul 151-741, Republic of Korea; ^6^Department of Ophthalmology, Chonnam National University Medical School and Hospital, Gwangju 501-757, Republic of Korea; ^7^Department of Molecular Physiology, College of Pharmacy, Kyungpook National University, Taegu 702-701, Republic of Korea

## Abstract

Oxidative stress-induced retinal pigment epithelial (RPE) cell damage is involved in the progression of diabetic retinopathy. Arginine methylation catalyzed by protein arginine methyltransferases (PRMTs) has emerged as an important histone modification involved in diverse diseases. Sirtuin (SIRT1) is a protein deacetylase implicated in the onset of metabolic diseases. Therefore, we examined the roles of type I PRMTs and their relationship with SIRT1 in human RPE cells under H_2_O_2_-induced oxidative stress. H_2_O_2_ treatment increased PRMT1 and PRMT4 expression but decreased SIRT1 expression. Similar to H_2_O_2_ treatment, PRMT1 or PRMT4 overexpression increased RPE cell damage. Moreover, the H_2_O_2_-induced RPE cell damage was attenuated by PRMT1 or PRMT4 knockdown and SIRT1 overexpression. In this study, we revealed that SIRT1 expression was regulated by PRMT1 but not by PRMT4. Finally, we found that PRMT1 and PRMT4 expression is increased in the RPE layer of streptozotocin-treated rats. Taken together, we demonstrated that oxidative stress induces apoptosis both via PRMT1 in a SIRT1-dependent manner and via PRMT4 in a SIRT1-independent manner. The inhibition of the expression of type I PRMTs, especially PRMT1 and PRMT4, and increased SIRT1 could be therapeutic approaches for diabetic retinopathy.

## 1. Introduction

Diabetic retinopathy is the leading cause of blindness. The breakdown of the blood-retinal barrier (BRB) mediated by oxidative stress is related to the progression of diabetic retinopathy [1, 2]. Retinal pigment epithelial (RPE) cells are a vital component of the outer BRB and are vulnerable to oxidative stress [3]. However, the molecular mechanisms of oxidative stress-induced RPE cell damage are not fully understood.

Protein arginine methyltransferases (PRMTs) catalyse the methylation of the arginine residues of histone and nonhistone proteins. Mammals possess nine PRMTs, which are divided into three types according to their method of methylation. Type 1 PRMTs (PRMT1, PRMT2, PRMT3, PRMT4, PRMT6, and PRMT8) catalyse asymmetric dimethylation at arginine residues, whereas type II PRMTs (PRMT5 and PRMT9) catalyse symmetric dimethylation, and type III PRMTs (PRMT7) catalyse monomethylation [4]. PRMT1 is thought to be involved in diabetic retinopathy, as PRMT1 expression is increased via the generation of reactive oxygen species (ROS) in the retinas of streptozotocin-treated rats and high-glucose-treated bovine retinal capillary endothelial cells, which are a crucial component of the inner BRB [5]. However, the regulation of PRMTs by oxidative stress in RPE cells has not been elucidated.

Sirtuin (SIRT1), a mammalian ortholog of yeast Sir2 (Silent Information Regulator 2), is an NAD-dependent histone deacetylase that regulates diverse physiological and pathophysiological processes, such as senescence, circadian rhythms, autophagy, and apoptosis [6]. In RPE cells, decreased SIRT1 expression caused by ultraviolet light is related to RPE cell damage [7]. The treatment of RPE cells with resveratrol, which increases SIRT1 activity, suppresses inflammatory cytokine-induced vascular endothelial growth factor (VEGF) secretion, which is involved in age-related macular degeneration (AMD) [8]. These reports suggest that SIRT1 protects against RPE cell dysregulation. However, the mechanisms regulating SIRT1 in RPE cells have not been evaluated.

In this study, we evaluated type I PRMT expression and SIRT1 expression under hydrogen peroxide- (H_2_O_2_-) induced oxidative stress and demonstrated that oxidative stress-induced PRMT1 expression increases RPE cell apoptosis via SIRT1 downregulation, whereas PRMT4 does so independently of SIRT1 expression.

## 2. Materials and Methods

### 2.1. Materials

Dulbecco's Modified Eagle's Medium (DMEM), Ham's nutrient mixture F-12, and fetal bovine serum (FBS) were purchased from Life Technologies (Gibco BRL, Grand Island, NY, USA). Hydrogen peroxide was obtained from Sigma-Aldrich (St. Louis, MO, USA). PRMT1 antibody (#2449), PRMT4 antibody (#4438), PARP1 antibody (#9532), and Caspase-3 antibody (#9662) were purchased from Cell Signaling Technology (Beverly, MA, USA). SIRT1 antibody (sc-15404) and *β*-actin antibody (sc-1616) were purchased from Santa Cruz Biotechnology (CA, USA). HA antibody (MMS-101R) was obtained from Covance (WI, USA). PRMT3 antibody was kindly provided by Mark T. Bedford (University of Texas, M. D. Anderson Cancer Center, Smithville, TX). All reagents were of the highest purity commercially available.

### 2.2. Cell Culture

The human RPE cell line ARPE-19 was obtained from the American Type Culture Collection (ATCC, Rockville, MD, USA). ARPE-19 cells were grown in DMEM/Ham's F-12 (1 : 1) supplemented with 10% fetal bovine serum (FBS) at 37°C in 5% CO_2_ in air. Stock cultures of ARPE-19 cells were subcultured once a week (split ratio 1 : 6). Cells were grown to confluence in 60 mm dishes in DMEM/Ham's F-12 with 15 mM HEPES buffer, 10% FBS, 5.5 mM glucose, 0.35% additional sodium bicarbonate, 2.5 mM L-glutamine, and 1% penicillin/streptomycin at 37°C. The media were changed every other day. Passaged cells were plated to yield near-confluent cultures at the end of the experiments.

### 2.3. MTT Assay

ARPE-19 cells were cultured on a 96-well plate in DMEM/Ham's F-12 (1 : 1) medium supplemented with 10% FBS. After treatments, the cells were treated with 500 *μ*g/mL of 3-(4,5-dimethysssl-thiazol-2-yl)-2,5-diphenyl tetrazolium bromide (MTT; Sigma) and incubated for 3 h in a CO_2_ incubator. Cells with a functional mitochondrial succinate dehydrogenase can convert MTT to formazan. The formazan crystals formed were solubilized in DMSO (Sigma) and measured with an ELx808 microplate spectrophotometer reader at *λ* = 570 nm (BioTek).

### 2.4. Western Blotting

Western blot analysis was performed according to methods described previously [9]. Transferred membranes were probed with various antibodies. The bands were visualized with Luminescent image analyzer (ImageQuant LAS 4000, GE Healthcare, UK) using Amersham ECL Western Blotting Detection Reagents (GE Healthcare, UK).

### 2.5. Plasmids and DNA Transfection

The Flag, Flag-SIRT1, and Flag-SIRT1 H363Y plasmids were kindly provided by Dr. Hueng-Sik Choi (School of Biological Sciences and Technology, Chonnam National University, Korea). HA, HA-PRMT1, and HA-PRMT4 were kindly provided by Dr. Fukamizu A (Life Science Center of Tsukuba Advanced Research Alliance, University of Tsukuba, Japan). The plasmids were transfected into ARPE-19 cells using PolyExpress transfection reagent (Excellgen, Gaithersburg, MD, USA) in accordance with the manufacturer's instructions.

### 2.6. siRNA Transfection

siRNA for PRMT1 (sc-41069; Santa Cruz Biotechnology, Santa Cruz, CA, USA), PRMT4 (sc-44875; Santa Cruz Biotechnology), and scramble siRNA (Qiagen, Hilden, Germany) were used to silence endogenous PRMT1 and PRMT4 expression. Lipofectamine RNAiMAX reagent (Invitrogen, Carlsbad, CA, USA) was used to transfect each siRNA (30 nM) following reverse transfection in accordance with the manufacturer's instructions.

### 2.7. Animal Experiments

Hyperglycemia was induced in overnight fasted, 10-week-old male SD rats (*n* = 7) by intraperitoneal injection of streptozotocin (55 mg/kg) dissolved in cold and fresh citrate buffer (0.1 M and pH 4.5). Control rats (*n* = 7) were injected with citrate buffer. Three days after STZ injection, plasma glucose level was determined after overnight fasting with Accu-Chek Aviva (Roche, Swiss). Rats with a blood glucose level of 300 mg/dL or higher were considered as diabetes. After 2 weeks, for preparation of cryosections, the rats were anaesthetized and eyeballs were enucleated, and then they were killed by CO_2_ inhalation. All animal experiments were performed in accordance with National Institutes of Health animal research standards. And protocols were approved by the Chonnam National University Laboratory Animal Research Center.

### 2.8. Immunohistochemistry (IHC) and Digital Image Analysis

Eyeballs were fixed in 4% paraformaldehyde in phosphate-buffered saline (PBS, pH 7.4) for 2 hours at 4°C. Eyeballs were frozen in OCT compound (Cellpath, Hemel Hempstead, UK). Cryosections of the retina (10 *μ*m) were cut through the optic nerve head. Fixation was performed with precooled (−20°C) acetone for 10 min. After allowing acetone to evaporate, immunostaining was performed according to the Vectastain ABC kit (Vector Labs; PK-6101). Briefly, endogenous peroxidase activity was quenched by 0.3% H_2_O_2_ treatment for 30 min and then sections were blocked with goat serum for 20 min and then probed with PRMT1 antibody (diluted 1 : 200) or PRMT4 antibody (diluted 1 : 300) for 60 min. After washing with PBS, sections were incubated with biotinylated secondary antibody for 30 min and then incubated with ABC reagent for 30 min. DAB (Vector Labs; SK-4100) was used for peroxidase substrate solution. Hematoxylin staining was performed for counter-staining. The immunohistochemistry-stained sections were observed using a BX-40 apparatus (Olympus, Tokyo, Japan) with an eXcope X3 digital camera (DIXI Optics, Daejeon, South Korea). Semiautomated analysis protocol was used to quantify the IHC images. Using imageJ, pure DAB images were deconvoluted from IHC images. The pixel intensities of pure DAB images were analyzed with histogram range from 0 to 255. The lower pixel value represents the higher positive signals of DAB. The pixel values were mainly spread from 111 to 200. The values between 111 and 140 were considered as high positive, 141–170 were positive, and 171–200 were low positive.

### 2.9. Statistical Analysis

The results were expressed as the mean ± SEM. Values are the mean ± SEM of three or four independent experiments. All the experiments were analyzed by analysis of variance (ANOVA). A *P* value < 0.05 was considered significant.

## 3. Results

### 3.1. H_2_O_2_ Increases PRMT1 and PRMT4 Expression and Decreases SIRT1 Expression

**Figure 1:**
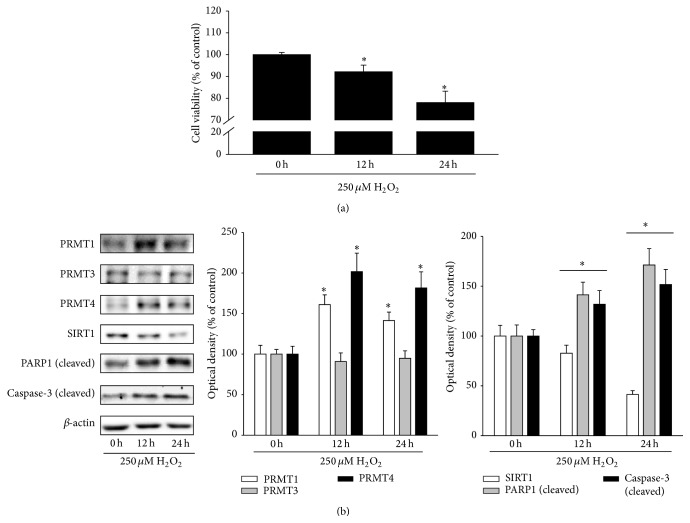
H_2_O_2_ increases PRMT1 and PRMT4 expression but decreases SIRT1 expression. (a, b) ARPE-19 cells were treated with 250 *μ*M H_2_O_2_ for 12 and 24 h. (a) Cell viability was measured by the MTT assay. The data represent the means ± SEM of three independent experiments, each performed in triplicate. ^*∗*^ *P* < 0.05 versus 0 h. (b) Cell extracts were subjected to Western blotting with the indicated antibodies. The data represent the means ± SEM of three independent experiments. ^*∗*^ *P* < 0.05 versus 0 h.

To induce oxidative stress, human retinal pigment epithelial cells (ARPE-19 cells) were treated with 250 *μ*M H_2_O_2_. As expected, H_2_O_2_ treatment decreased cell viability and increased the cleavage of PARP1 and caspase-3, which are associated with RPE cell apoptosis (Figures 1(a) and 1(b)) [10]. Furthermore, H_2_O_2_ treatment increased PRMT1 and PRMT4 expression and decreased SIRT1 expression, while PRMT3 expression was unchanged (Figure 1(b)).

### 3.2. SIRT1 Overexpression Attenuates Oxidative Stress-Induced RPE Cell Apoptosis

**Figure 2:**
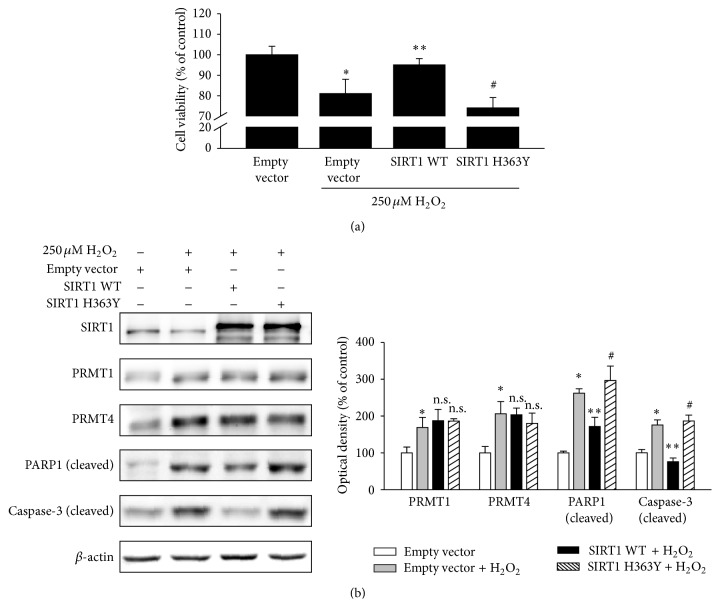
SIRT1 overexpression attenuates oxidative stress-induced RPE cell apoptosis. (a, b) ARPE-19 cells were transfected with empty vector, SIRT1 WT, or SIRT1 H363Y plasmids. After 24 h, the medium was changed to serum-free medium and 250 *μ*M H_2_O_2_ was added for 24 h. (a) Cell viability was measured by the MTT assay. The data represent the means ± SEM of three independent experiments, each performed in triplicate. ^*∗*^ *P* < 0.05 versus empty vector, ^*∗∗*^ *P* < 0.05 versus empty vector + 250 *μ*M H_2_O_2_, and ^#^ *P* < 0.05 versus SIRT1 WT + 250 *μ*M H_2_O_2_. (b) Cell extracts were subjected to Western blotting with the indicated antibodies. The data represent the means ± SEM of three independent experiments. ^*∗*^ *P* < 0.05 versus empty vector, ^*∗∗*^ *P* < 0.05 versus empty vector + 250 *μ*M H_2_O_2_, and ^#^ *P* < 0.05 versus SIRT1 WT + 250 *μ*M H_2_O_2_ (n.s. = nonspecific).

As H_2_O_2_ treatment decreased SIRT1 expression, we postulated that oxidative stress-induced RPE cell damage is regulated by SIRT1. To confirm this, ARPE-19 cells were transfected with empty vector or SIRT1 followed by H_2_O_2_. SIRT1 overexpression restored the cell viability lowered by H_2_O_2_ treatment (Figure 2(a)). Moreover, the increased cleavage of PARP1 and caspase-3 was attenuated by SIRT1 overexpression (Figure 2(b)). However, transfection of SIRT1 H363Y, an enzymatic-dead mutant that lacks deacetylase activity because histidine 363 is converted to tyrosine [11], did not restore cell viability or attenuate PARP1 and caspase-3 cleavage (Figures 2(a) and 2(b)). SIRT1 or SIRT1 H363Y overexpression did not influence PRMT1 or PRMT4 expression under oxidative stress (Figure 2(b)).

### 3.3. PRMT1 or PRMT4 Overexpression Increases RPE Cell Apoptosis and PRMT1 Overexpression Decreases SIRT1 Expression

**Figure 3:**
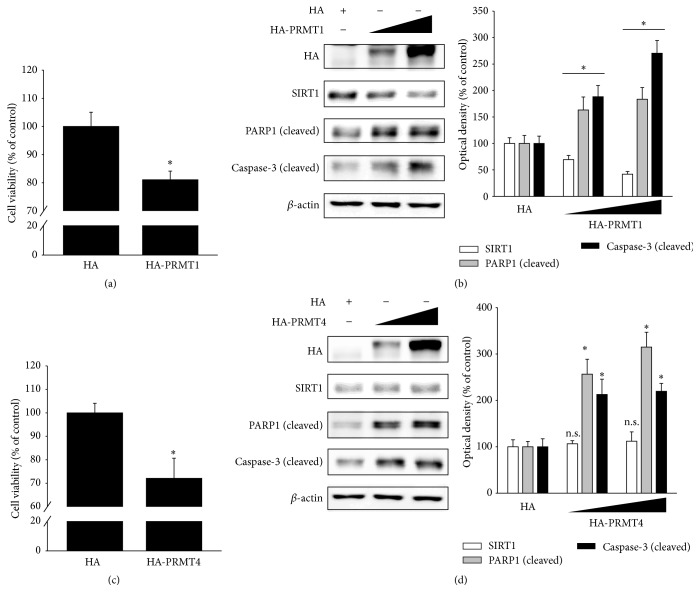
PRMT1 or PRMT4 overexpression increases RPE cell apoptosis, while PRMT1 overexpression decreases SIRT1 expression. (a, b) ARPE-19 cells were transfected with HA or HA-PRMT1 plasmid DNA. After 36 h, (a) cell viability was measured by the MTT assay. The data represent the means ± SEM of three independent experiments, each performed in triplicate. ^*∗*^ *P* < 0.05 versus HA. (b) Cell extracts were subjected to Western blotting with the indicated antibodies. The data represent the means ± SEM of three independent experiments. ^*∗*^ *P* < 0.05 versus HA. (c, d) ARPE-19 cells were transfected with HA or HA-PRMT4 plasmid DNA. After 36 h, (c) cell viability was measured by the MTT assay. The data represent the means ± SEM of three independent experiments, each performed in triplicate. ^*∗*^ *P* < 0.05 versus HA. (d) Cell extracts were subjected to Western blotting with the indicated antibodies. The data represent the means ± SEM of three independent experiments. ^*∗*^ *P* < 0.05 versus HA (n.s. = nonspecific).

H_2_O_2_ treatment increased PRMT1 and PRMT4 expression (Figure 1(b)). To determine the effect of the increased PRMT1 or PRMT4 on RPE cell apoptosis, ARPE-19 cells were transfected with HA, HA-PRMT1, or HA-PRMT4. PRMT1 overexpression decreased cell viability and SIRT1 expression (Figures 3(a) and 3(b)). Moreover, PRMT1 overexpression increased the cleavage of PARP1 and caspase-3 (Figure 3(b)). PRMT4 overexpression also decreased cell viability and increased the cleavage of PARP1 and caspase-3 but did not alter SIRT1 expression (Figures 3(c) and 3(d)).

### 3.4. PRMT1 or PRMT4 Knockdown Attenuates Oxidative Stress-Induced RPE Cell Damage and PRMT1 Expression Regulates SIRT1 Expression

**Figure 4:**
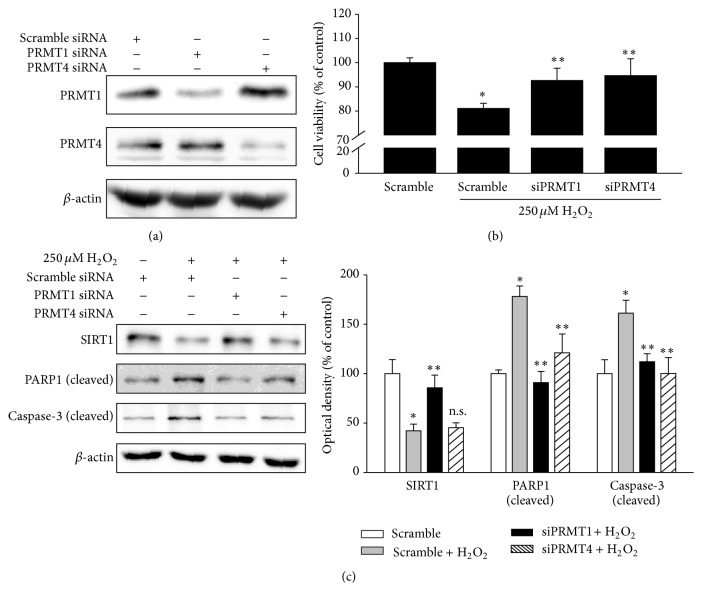
The knockdown of PRMT1 or PRMT4 attenuates oxidative stress-induced RPE cell damage and PRMT1 expression regulates SIRT1 expression. (a) ARPE-19 cells were transfected with scramble, PRMT1, or PRMT4 siRNA according to the reverse transfection method. After 36 h, cell extracts were subjected to Western blotting with the indicated antibodies. (b, c) ARPE-19 cells were transfected with scramble, PRMT1, or PRMT4 siRNA according to the reverse transfection method. After 24 h, the medium was changed to serum-free medium and 250 *μ*M H_2_O_2_ was added for 24 h. (b) Cell viability was measured by the MTT assay. The data represent the means ± SEM of three independent experiments, each performed in triplicate. ^*∗*^ *P* < 0.05 versus scramble siRNA, ^*∗∗*^ *P* < 0.05 versus scramble siRNA + 250 *μ*M H_2_O_2_. (c) Cell extracts were subjected to Western blotting with the indicated antibodies. The data represent the means ± SEM of three independent experiments. ^*∗*^ *P* < 0.05 versus scramble siRNA, ^*∗∗*^ *P* < 0.05 versus scramble siRNA + 250 *μ*M H_2_O_2_ (n.s. = nonspecific).

To confirm these findings, PRMT1 or PRMT4 expression was silenced by siRNA transfection (Figure 4(a)). PRMT1 or PRMT4 knockdown restored the H_2_O_2_-induced decrease in cell viability (Figure 4(b)). Moreover, the increased cleavage of PARP1 and caspase-3 was attenuated by knockdown of PRMT1 or PRMT4 (Figure 4(c)). The H_2_O_2_-induced SIRT1 downregulation was restored by PRMT1 knockdown but not by PRMT4 knockdown (Figure 4(c)).

### 3.5. PRMT1 and PRMT4 Expression Is Increased in the RPE Layer of Streptozotocin-Treated Rats

**Figure 5:**
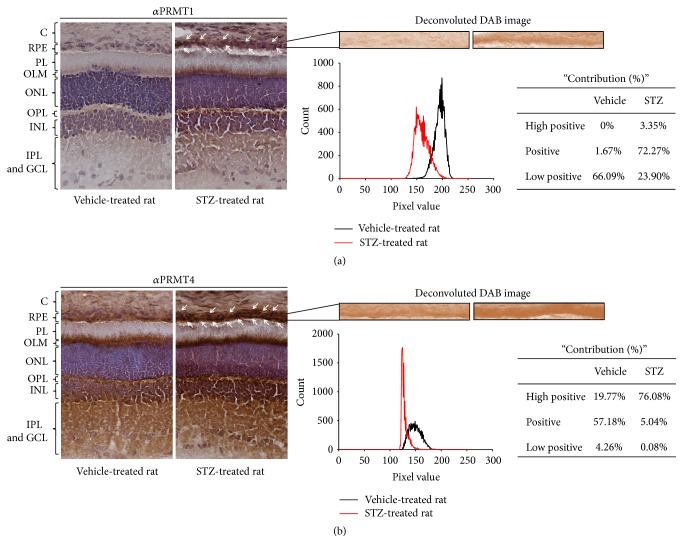
PRMT1 and PRMT4 expression is increased in the RPE layer of streptozotocin-treated rats. Eyeballs were enucleated from vehicle-treated and STZ-treated rats and cryosections were prepared. (a, b) PRMT1 (a) and PRMT4 (b) expressions were measured by immunohistochemistry analysis (C: choroid, RPE: retinal pigment epithelium, PL: photoreceptor layer, OLM: outer limiting membrane, ONL: outer nuclear layer, OPL: outer plexiform layer, INL: inner nuclear layer, IPL: inner plexiform layer, and GCL: ganglion-cell layer). Representative images were from at least three independent experiments. To quantify the DAB signaling, semiautomated analysis protocol was performed as described in Section 2.

To confirm the increase of PRMT1 and PRMT4 expression* in vivo*, we generated rats with streptozotocin- (STZ-) induced diabetes, which show severe hyperglycemia and are known to have induced diabetic retinopathy via oxidative stress [12, 13]. As shown in Figures 5(a) and 5(b), PRMT1 and PRMT4 expression was significantly increased in the RPE layer of STZ rats compared with vehicle-treated rats (white arrows). Furthermore, high positive and positive signals of PRMT1 and high positive signals of PRMT4 were greatly increased in STZ rats compared with vehicle-treated rats (Figures 5(a) and 5(b)).

## 4. Discussion

Type I PRMTs are important pathophysiological regulators as they promote the production of asymmetric dimethylarginine (ADMA), a metabolic by-product that inhibits nitric oxide synthase (NOS), which is involved in cardiovascular disease, diabetes, and other metabolic disorders [14–16]. In addition to producing ADMA, type I PRMTs regulate various cellular processes, such as transcription, RNA splicing, and signal transduction, by catalysing the asymmetric dimethylation of histone or nonhistone proteins [17]. Recent studies have revealed the role of type I PRMTs in diabetic nephropathy [18, 19]. However, their roles in diabetic retinopathy are rarely known. In this study, we demonstrated that increased expression of the type I PRMTs, PRMT1 and PRMT4, induced RPE cell damage under oxidative stress. This was supported by the following evidence: (1) H_2_O_2_ treatment increased not only RPE cell damage but also PRMT1 and PRMT4 expression; (2) PRMT1 or PRMT4 overexpression increased RPE cell damage; (3) PRMT1 or PRMT4 knockdown attenuated the H_2_O_2_-induced RPE cell damage; and (4) PRMT1 and PRMT4 expression was increased in the RPE layer of STZ-treated rats.

Many studies have demonstrated that oxidative stress-induced cellular damage is mediated by increased type I PRMT expression. For example, H_2_O_2_-induced oxidative stress increases PRMT3 expression, leading to increased ADMA generation in preglomerular vascular smooth muscle cells [20]. Treatment with human serum albumin, which induces oxidative stress in renal proximal tubular epithelial cells, increased PRMT1 expression [21]. In contrast, few studies have revealed the protective effects of type I PRMTs in oxidative stress. Very recently, Huang et al. reported that arsenic-induced oxidative stress recruits PRMT1 to the histone 4 arginine 3 and PRMT4 to the histone 3 arginine 17 for asymmetric dimethylation, which leads to increased ferritin transcription via the antioxidant responsive element in HaCaT cells (human keratinocytes) [22]. In the report by Huang et al., in contrast to our results, the expression of PRMT1 and PRMT4 was not changed and PRMT1 and PRMT4 increased ferritin to protect cells from oxidative stress. This discrepancy was likely due to the different cell types (retinal pigment epithelial cells versus keratinocyte) or the kind of oxidative stress (H_2_O_2_ versus arsenic), as arsenic treatment induces superoxide anion (O_2_^∙−^) and hydroxyl radical (^*∙*^OH) production [23]. Moreover, PRMT1 and PRMT4 were examined within 6 h of arsenic treatment, while our experiment involved a relatively long duration of H_2_O_2_ treatment. Wang et al. reported that treatment with lithium and valproate acid, which protect against H_2_O_2_-induced oxidative stress, increase PRMT4 expression in NSC34 cells [24]. However, they did not establish the function of PRMT4.

In this study, we also showed that H_2_O_2_-induced SIRT1 downregulation is involved in RPE cell damage. Several lines of evidence support our findings. Wu et al. reported that SIRT1 overexpression reverses H_2_O_2_-mediated complement factor H downregulation in ARPE-19 cells [25]. Moreover, Bhattacharya et al. reported that decreased SIRT1 expression in RPE cells induces p53 acetylation-mediated apoptosis, leading to the progression of age-related macular degeneration (AMD) [26]. Indeed, p53 and its target genes are closely involved in RPE cell apoptosis [27, 28]. As a deacetylase, SIRT1 inhibits p53 activity via deacetylation at lysine 382 [29]. In our study, transfection with an enzymatic-dead mutant, SIRT1, did not inhibit the H_2_O_2_-induced RPE cell apoptosis. Here, we provide novel evidence that SIRT1 expression and its enzymatic activity are vital for RPE cell maintenance.

Interestingly, we found that SIRT1 expression is negatively regulated by PRMT1 but not by PRMT4. Scalera et al. reported that red wine decreased PRMT1 expression in a SIRT1-dependent manner in human endothelial cells [30]. However, in our study, SIRT1 overexpression did not alter PRMT1 expression. This may be due to a cell-type-specific response (endothelial versus epithelial cells). We speculated that PRMT4 regulates SIRT1 expression, as PRMT4 increases the stability of SIRT1 mRNA by methylating HuR protein at arginine 217 in stem cells and HeLa cells [31, 32]. However, PRMT4 did not influence SIRT1 protein expression in ARPE-19 cells. To our knowledge, this is the first report on the relationship between PRMT and SIRT1 in cell function. We provide the first evidence that PRMT1 regulates SIRT1 under oxidative stress.

Signaling induced by high-glucose is highly associated with oxidative stress [33]. Very recently, we reported that PRMT4 expression is increased by high-glucose in RPE cells [34]. Consistent with previous results, PRMT4 expression was increased in the RPE and outer limiting membrane (OLM) layers of STZ rats. In addition, PRMT1 expression was increased in the RPE and overall layers of retina of STZ rats. It may be speculated that increased PRMT1 and PRMT4 expression in other layers by STZ treatment contributes to the progression of retinopathy. Further studies should be performed to reveal this speculation.

**Figure 6:**
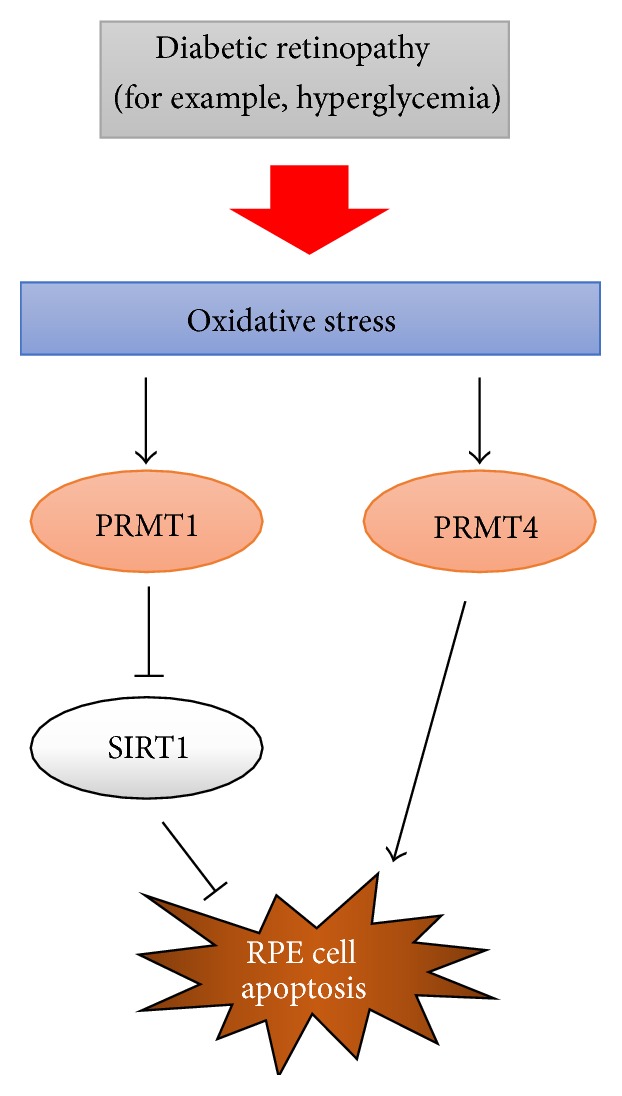
PRMT1 and PRMT4 regulate oxidative stress-induced RPE cell damage in SIRT1-dependent and SIRT1-independent manners.

In this study, we demonstrated that oxidative stress-induced RPE cell damage is regulated by type I PRMTs (PRMT1 and PRMT4) and oxidative stress-induced SIRT1 downregulation is involved in the PRMT1-mediated RPE cell apoptosis pathway. Oxidative stress is a major cause of diabetic retinopathy. Taken together, our data suggest a model of signaling pathways involved in oxidative stress-induced RPE cell apoptosis (Figure 6). Therefore, the inhibition of type I PRMT expression, especially PRMT1 and PRMT4, and the increase in SIRT1 expression could be therapeutic approaches for diabetic retinopathy.
